# Phylogenomic insight into dysploidy, speciation, and plastome evolution of a small Mediterranean genus *Reichardia* (Cichorieae; Asteraceae)

**DOI:** 10.1038/s41598-022-15235-1

**Published:** 2022-06-30

**Authors:** Myong-Suk Cho, JiYoung Yang, José A. Mejías, Seung-Chul Kim

**Affiliations:** 1 Department of Biological Sciences, Sungkyunkwan University, Suwon, 16419 Republic of Korea; 2 Research Institute for Dok-do and Ulleung-do Island, Kyungpook National University, Daegu, 41566 Republic of Korea; 3 Department of Plant Biology and Ecology, University of Seville, Seville, Spain

**Keywords:** Evolution, Plant sciences

## Abstract

*Reichardia* Roth is a small Mediterranean genus comprising ten homogeneous species with basic chromosome numbers of 7, 8, and 9. To assess the plastid genome evolution and differentiation of *Reichardia* species, we assembled the complete plastome sequences of seven *Reichardia* and two *Launaea* species and conducted various phylogenomic analyses comparatively with nuclear ribosomal DNA ITS sequences. *Reichardia* and *Launaea* plastomes were highly conserved in gene content and order, containing 130 genes. Plastid phylogenomic reconstruction strongly suggested that *Reichardia* was a sister to *Launaea*, and its common ancestor initially diverged into two major lineages: the first containing species with n = 8 chromosomes exclusively, and the other with n = 9, 8, and 7 chromosomes. Although the ancestral *Reichardia* karyotype was suggested to most likely be n = 9 from ancestral chromosome number reconstruction, the pattern of descending dysploidy indicated by the phylogenetic trees based on nuclear ribosomal DNA ITS was less evident in the trees based on the plastome. Possible reasons for these findings are discussed.

## Introduction

**Figure 1:**
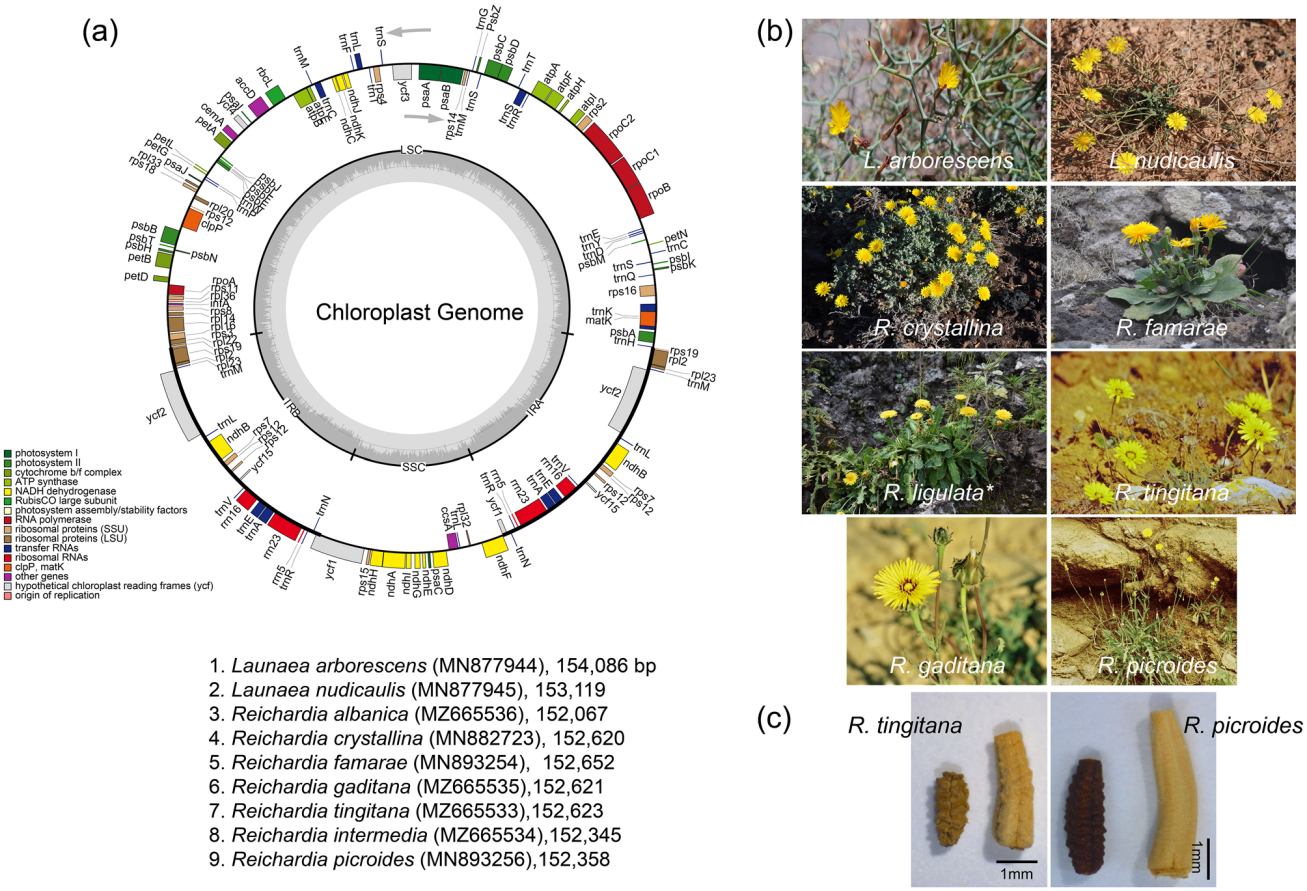
(**a**) Gene map of the chloroplast genomes of seven *Reichardia* and two *Launaea* species sequenced in this study. The genes inside and outside of the circle are transcribed in the clockwise and counterclockwise directions, respectively. Genes belonging to different functional groups are shown in different colors. The thick lines indicate the extent of the inverted repeats that separate the genomes into small single copy (SSC) and large single copy (LSC) regions. (**b**) Photos of *Reichardia* and *Launaea* species. (**c**) Photos of outer (left) and inner (right) heteromorphic achenes in *R. tingitana* and *R. picroides*. Photo credit: José A. Mejías (*L. nudicaulis, R. gaditana, R. tingitana, R. picroides*, and fruits), Myong-Suk Cho (*L. arborescens*), Arnoldo Santos-Guerra (*R. crystallina*), and Seung-Chul Kim (*R. famarae* and *R. ligulata*).

*Reichardia* is a small genus in the subtribe Hyoseridinae Less. (formerly known as Sonchinae) from the tribe Cichorieae Lam. & DC. (Asteraceae). It comprises ten herbaceous species, of which seven are perennials with woody bases [*R. dichotoma* (DC.) Freyn, *R. macrophylla* (Vis. & Pančić) Pančić, *R. albanica* F. Conti & D. Lakušić, *R. picroides* (L.) Roth, *R. crystallina* (Sch. Bip.) Bramwell, *R. famarae* Bramwell & G. Kunkel, and *R. ligulata* (Vent.) G. Kunkel and Sunding]; a biennial-perennial cycle [*R. gaditana* (Willk.) Samp.]; and two are annuals [*R. tingitana* (L.) Roth and *R. intermedia* (Sch. Bip.) Samp.]. Although all of these are native to the Mediterranean and/or Macaronesia (North Atlantic), those with wider distribution, namely *R. tingitana* and *R. picroides*, also colonize Middle East Asia. In addition, *R. tingitana* reaches Pakistan, northwestern India, and some areas of tropical and subtropical eastern Africa, showing a range roughly coinciding with the paleo-geographical limits of the Mediterranean or ‘Madrean-Tethyan’ region^1,2^. These two widely distributed species were introduced in Hawaii and Australia^3–6^. *R. intermedia* is also a circum-Mediterranean taxon but is quite common in the western area. *R. gaditana* is limited to the western Mediterranean region, whereas *R. dichotoma* is exclusively present in the eastern Mediterranean region. Five species show endemic distributions: the close relatives *R. macrophylla* and *R. albanica*, which are restricted to the Balkan Peninsula^7^; and *R. crystallina*, *R. famarae*, and *R. ligulata*, which are endemic to the Canary Islands. *R. famarae* occurs exclusively in five localities in Lanzarote and Fuerteventura^8,9^. The genus *Reichardia* is quite homogeneous in morphology and is easily recognizable (Fig. 1), mainly by the flower heads, which are often large and typically broadly conical when fruiting, and heteromorphic fruits. Outer and medium phyllaries show typical scarious margins and a subterminal mucro. Although florets are generally yellow, in two species (*R. tingitana* and *R. gaditana*), the base of the ligule is deep purple. Achenes are four-ribbed, outer transversely rugose, while compressed inner ones are paler and variably smooth. The pappus consists of numerous fine scabrid bristles.

Despite its low species diversity, *Reichardia* presents three basic chromosome number groups of n = 7, 8, and 9^3,10–16^. All the species are diploid, and no cases of polyploidy have been documented. The chromosome numbers n = 9 and 2n = 18 have been reported for the Western Asiatic *R. dichotoma* and the Balkan *R. macrophylla*, which can be considered vicarious species^7^ and perhaps tertiary relicts^17^. The recently described Albanian endemic *R. albanica* also showed a chromosome number of 2n = 18. Several chromosomes count in the widespread species *R. tingitana*, *R. gaditana*, and *R. intermedia* have shown n = 8 and 2n = 16, which are also the numbers found in the three species endemic to the Canary Islands, namely *R. crystallina*, *R. famarae*, and *R. ligulata*. *R. picroides* showed chromosome numbers of n = 7 and 2n = 14. Although some authors have reported a somatic number of 2n = 14 for *R. intermedia*^11,18,19^ in samples from the Iberian Peninsula, we chose to apply the somatic numbers from a monographic study of the genus by Gallego^13^ based on their subsequent reconfirmation from the Iberian materials^15^.

The diversity observed in chromosome numbers in the genus is presumed to be the result of dysploidy during karyotypic changes in the basic chromosome number^19–22^, as no polyploidy numbers have ever been reported for them, despite it being a widespread phenomenon reported in Asteraceae^23^. Previous studies have shown a correlation between this diversity and morphological characteristics^6^, chemical compounds^24^, and phylogenetic lineages^19^, claiming both ascending and descending patterns of dysploidy in *Reichardia*. Exceptional chromosomal diversity in *Reichardia* has been the subject of evolutionary studies as a good model for the analysis of genome evolution and differentiation. For example, Lӧve and Kjellqvist^11^ suggested that the most primitive number was n = 7, and the variation from primitive to higher chromosome numbers (for example, n = 8) was derived by means of the appearance of B chromosomes. However, Gallego^13^ proposed an alternative hypothesis that the primitive basic chromosome number was n = 9, and the secondary basic numbers of n = 8 and n = 7 arose from the loss of chromosomes or fragmentation. The analysis of phenolic compounds in *R. tingitana* and *R. picroides* did not support any specific pattern, neither ascending nor descending, but found that chemo-taxonomic differences related to morphological and karyological characteristics were in agreement with the separation of the *Reichardia* species into two major groups^24^. Recently, the chromosomal and genomic evolution of *Reichardia* species has been investigated within a molecular phylogenetic framework. Siljak-Yakovlev et al.^19^ plotted the karyological, cytogenetic, and pollen traits of *Reichardia* species onto the nuclear ribosomal DNA (nrDNA) internal transcribed spacer (ITS) phylogeny and suggested that the descending dysploidy in *Reichardia* chromosomal numbers was accompanied by changes in heterochromatin patterns and modifications of the location and organization of ribosomal genes. They also reported smaller pollen sizes in *R. picroides* and *R. intermedia*, which correlated with their smaller genome sizes and the reduced chromosome number within the genus (n = 7 for both species); however, in our view, these reports for *R. intermedia* may be erroneous. Moreover, these cytological studies did not include appropriate closest relatives or progenitors of the genus. Without robust phylogenetic relationships between *Reichardia* and other closely related genera and precise species relationships within the genus, it may be unsuitable to explore genome evolution and differentiation.

The phylogenetic position of *Reichardia* was previously inferred using nrDNA ITS and chloroplast DNA (cpDNA) *psbA-trnH* intergenic spacer and *matK* gene sequences^25–28^. In all phylogenies, the genera *Launaea* and *Reichardia* represented early diverging lineages within the subtribe Hyoseridinae, and *Reichardia* was monophyletic, which is concordant with the first comprehensive molecular analyses of the tribe Cichorieae^29^. The ITS tree strongly supported the monophyly of *Reichardia*^25,26,28^, which was sister to the clade containing the genus *Launaea* and the remaining genera of the subtribe Hyoseridinae, that is, the currently circumscribed *Sonchus* sensu lato (s.l.). The cpDNA trees based on either the *psbA-trnH* intergenic spacer^27^ or *matK*^28^ gene regions also supported the monophyly of *Reichardia* and the basal position of *Reichardia* and *Launaea*. However, cpDNA phylogenies in previous studies^27,28^ were too limited to determine the phylogenetic position of *Reichardia* and robust species relationships due to insufficient molecular markers and species representation. The relationships between species and genera were either highly unresolved or weakly supported, specifically in the *psbA-trnH* tree^27^. Unlike the nrDNA ITS-based phylogeny, *Reichardia* and *Launaea* shared the most recent common ancestor, and these two genera were sister to the remaining genera of the subtribe Hyoseridinae, which was also weakly supported (< 50% in the *psbA-trnH* tree)^27^ or moderately supported (86% in the *matK* tree)^28^. These conclusions are consistent with subsequent studies based on ITS datasets^19,30^.

With the advent of high-throughput sequencing technologies for next-generation sequencing (NGS), massive amounts of data have now become available based on the considerable genome-wide variation of entire plastid genomes through plastome sequencing. The benefits of genome-wide variation could increase phylogenetic resolution tremendously and significantly enhance our understanding of plant evolution and diversity in the field of plastid genetics and genomics^31^. Comparative genomic analysis using whole plastome sequences is now an efficient option to improve phylogenetic resolution at lower taxonomic levels that are currently hindered by limited sequence variation due to recent divergence, rapid radiation, and conservative genome evolution of plastomes^32^.

In this study, we sequenced and assembled the whole plastid genomes of seven *Reichardia* species in addition to *R. ligulata*, sequenced in our previous study^33^. The plastomes of two additional species from the closely related genus *Launaea* were also sequenced^25–28^. Based on these complete plastome sequences, we first tested the previous phylogenetic hypotheses proposed by nrDNA ITS and cpDNA markers; plastome-based phylogeny was reconstructed using genome-wide variation and compared with the nrDNA ITS phylogeny, newly reconstructed in this study. Second, through the ancestral karyotype reconstruction, we inferred the karyotype evolution in the dysploidy process of *Reichardia* species in the framework of the plastome and nrDNA ITS-based phylogenies. We also performed several comparative plastome analyses to determine the structure, gene content, and rearrangements of the plastid genomes of *Reichardia* and two *Launaea* species. In addition, we identified highly variable plastid regions that could be utilized as useful markers for further population genetic or phylogeographic studies of *Reichardia* and closely related genera.

## Results

### Genome features, content, order, and organization of *Reichardia* plastomes

**Figure 2:**
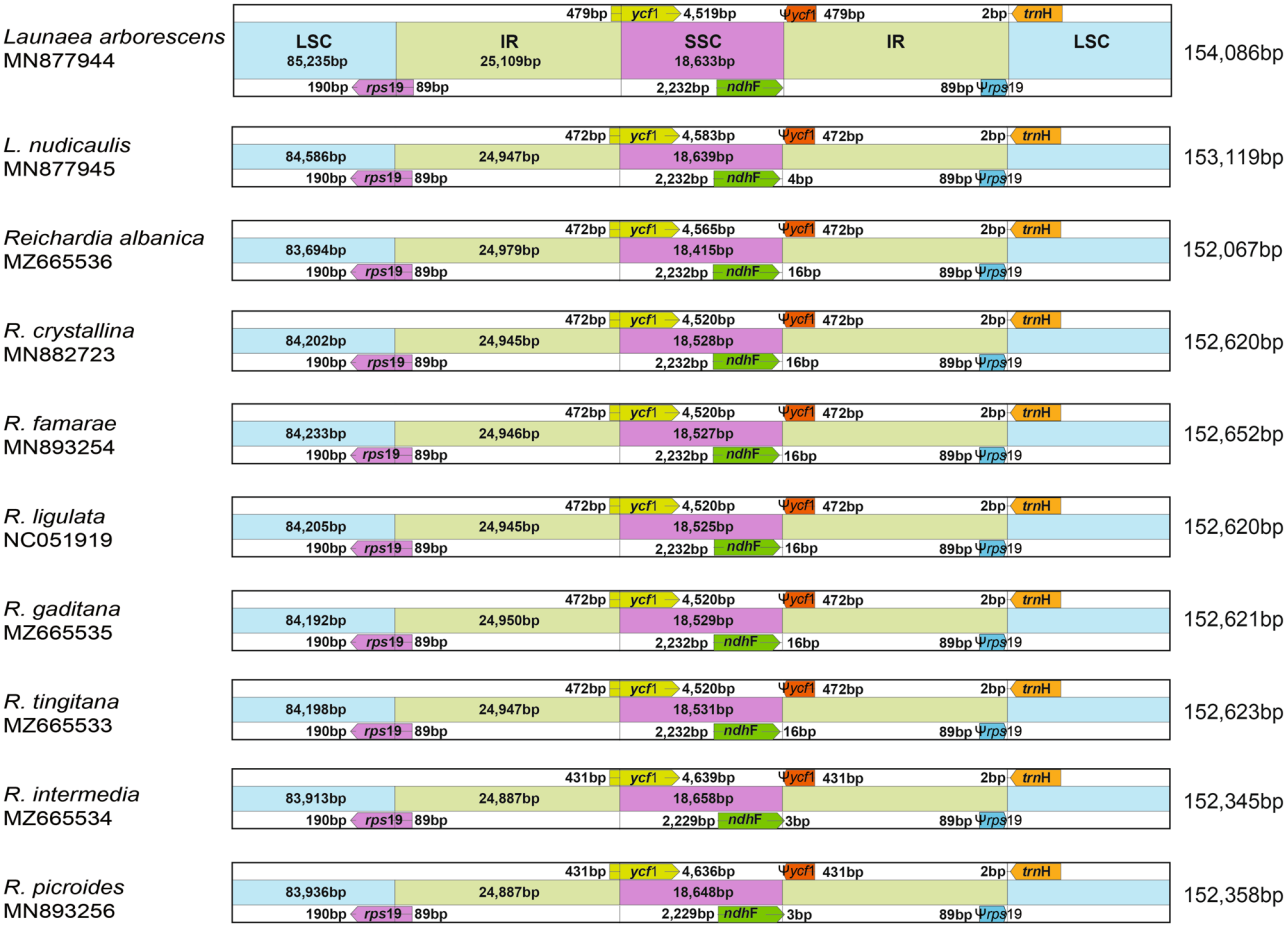
Comparison of the border positions of the large single copy (LSC), small single copy (SSC), and inverted repeat (IR) regions among eight *Reichardia* and two *Launaea* plastomes.

The eight plastomes of *Reichardia* species (*R. albanica, R. crystallina, R. famarae, R. gaditana, R. intermedia, R. ligulata, R. picroides*, and *R. tingitana*) and two species of *Launaea* (*L. arborescens* and *L. nudicaulis*) were highly conserved in terms of gene content and arrangement, with 98.6% pairwise sequence similarity (99.2% among eight *Reichardia* species only) (Table 1). The total length of eight *Reichardia* plastomes ranged from 152,067 (*R. albanica*) to 152,652 (*R. famarae*) base pairs (bp) with an overall guanine-cytosine (GC) content of 37.6%, whereas two plastomes of *Launaea* were slightly longer than *Reichardia* plastomes, that is, 153,119 bp for *L. nudicaulis* and 154,086 bp for *L. arborescens* with an overall GC content of 37.4%. The plastomes of *Reichardia* and *Launaea* species consisted of four typical regions: LSC, SSC, and a pair of inverted repeat regions (IRs), sharing exactly the same genes and similar gene contents at all boundaries among the four regions, with slight changes in the length of intergenic regions. They all contained the functional protein-coding gene *ycf1* at SSC/IR with its pseudogene copy, *ycf1*Ψ at IR/SSC, and functional *rps19* at LSC/IR with pseudogene copy *rps19*Ψ at IR/LSC endpoints (Fig. 2).

**Table 1:** Summary of the genomic characteristics of two *Launaea* and eight *Reichardia* chloroplast genomes used for comparative genomic analyses in this study.

Species GenBank accession no	Voucher (Herbarium)	Total size (bp) GC content (%)	LSC (bp) GC content (%)	IR (bp) GC content (%)	SSC (bp) GC content (%)	No. of total genes	No. of protein coding genes	No. of tRNA genes	No. of rRNA genes
**Genus *Launaea***									
*L. arborescens* MN877944	*Kim *et al*. 1040* (OS)	154,086 37.4%	85,235 35.5%	25,109 43.0%	18,633 31.2%	130	87	37	6
*L. nudicaulis* MN877945	*Kim *et al*. 1053* (OS)	153,119 37.5%	84,586 35.7%	24,947 43.1%	18,639 31.1%	130	87	37	6
**Genus *Reichardia***									
*R. albanica* MZ665536	*APP 56321* (APP)	152,067 37.6%	83,694 35.8%	24,979 43.0%	18,415 31.2%	130	87	37	6
*R. crystallina* MN882723	*Santos-Guera & Kim 210 (OS)*	152,620 37.6%	84,202 35.7%	24,945 43.1%	18,528 31.1%	130	87	37	6
*R. famarae* MN893254	*Santos-Guera & Kim 211* (OS)	152,652 37.6%	84,233 35.7%	24,946 43.1%	18,527 31.1%	130	87	37	6
*R. ligulata*^a^ NC051919	*Kim *et al*. 1044* (OS)	152,620 37.6%	84,205 35.7%	24,945 43.1%	18,525 31.1%	130	87	37	6
*R. gaditana* MZ665535	*Mejías JA SEV289342* (SEV)	152,621 37.6%	84,192 35.7%	24,950 43.1%	18,529 31.1%	130	87	37	6
*R. tingitana* MZ665533	*Mejías JA SEV289341* (SEV)	152,623 37.6%	84,198 35.7%	24,947 43.1%	18,531 31.1%	130	87	37	6
*R. intermedia* MZ665534	*Mejías JA SEV289340* (SEV)	152,345 37.6%	83,913 35.8%	24,887 43.1%	18,658 31.2%	130	87	37	6
*R. picroides* MN893256	*Jansen 2873* (TEX)	152,358 37.6%	83,936 35.8%	24,887 43.1%	18,648 31.1%	130	87	37	6

Herbarium code: APP: Parco Nazionale del Gran Sacco e Mont della Laga-Universita di Camerino, Italy, OS: Ohio State University herbarium, SEV: Herbario de la Universidad de Sevilla, TEX: University of Texas Herbarium.

^a^Sequenced from a previous study^33^. The remaining nine cp genomes were sequenced for this study.

Each of the eight *Reichardia* and two *Launaea* cp genomes contained 130 genes, including 87 protein-coding genes (excluding pseudogenes), six rRNA genes, and 37 tRNA genes (Tables 1 and 2). Twenty-four genes contained introns, including nine tRNA genes. Three genes, *clpP*, *rps12*, and *ycf3*, had two introns. The *trnK*-UUU tRNA gene harbors the largest intron, which contains the *matK* gene. In total, 17 genes were duplicated in the IR regions, including seven tRNAs, three rRNAs, and seven protein coding genes. The trans-splicing gene *rps12*, consisting of three exons, was located in the LSC region of exon 1, whereas exons 2 and 3 of the gene were embedded in the IR regions. Parts of *ycf1* and *rps19* duplicated in the IR region were considered pseudogenes in all cp genomes sequenced in this study.

**Table 2:** Genes encoded by two *Launaea* and eight *Reichardia* chloroplast genomes.

Category	Group	Genes
Photosynthesis	Subunits_of_photosystem_I	*psa*A, *psa*B, *psa*C, *psa*I, and *psa*J
	Subunits_of_photosystem_II	*psb*A, *psb*B, *psb*C, *psb*D, *psb*E, *psb*F, *psb*H, *psb*I, *psb*J, *psb*K, *psb*L, *psb*M, *psb*N, *psb*T, and *psb*Z
	Subunits_of_NADH_dehydrogenase	*ndh*A*, *ndh*B($\times$2)*, *ndh*C, *ndh*D, *ndh*E, *ndhF*, *ndh*G, *ndh*H, *ndh*I, *ndh*J, and *ndh*K
	Subunits_of_cytochrome_b/f_complex	*pet*A, *pet*B*, *pet*D*, *pet*G, *pet*L, and *pet*N
	Subunits_of_ATP_synthase	*atp*A, *atp*B, *atp*E, *atp*F*, *atp*H, and *atp*I
	Large_subunit_of_Rubisco	*rbc*L
Self-replication	Large_subunits_of_ribosome	*rpl*2($\times$2)*, *rpl*14, *rpl*16*, *rpl*20, *rpl*22, *rpl*23($\times$2), *rpl*32, *rpl*33, and *rpl*36
	Small_subunits_of_ribosome	*rps*2, *rps*3, *rps*4, *rps*7($\times$2), *rps*8, *rps*11, *rps*12 ($\times$2)**, *rps*14, *rps*15, *rps*16*, *rps*18, and *rps*19
	DNA-dependent_RNA_polymerase	*rpo*A, *rpo*B, *rpo*C1*, and *rpo*C2
	translation initiation factor	*inf*A
	Ribosomal_RNAs	*rrn*5($\times$2), *rrn*16($\times$2), and *rrn*23($\times$2)
	Transfer_RNAs	*trn*A-UGC($\times$2)*, *trn*C-GCA, *trn*D-GUC, *trn*E-UUC($\times$3)*, *trn*F-GAA, *trn*G-GCC, *trn*H-GUG, *trn*K-UUU*, *trn*L-CAA($\times$2)*, *trn*L-UAA, *trn*L-UAG, *trn*M-CAU($\times$4), *trn*N-GUU($\times$2), *trn*P-UGG, *trn*Q-UUG, *trn*R-ACG($\times$2), *trn*R-UCU, *trn*S-CGA, *trn*S-GCU*, *trn*S-GGA, *trn*S-UGA, *trn*S-GGU, *trn*T-UGU, *trn*V-GAC($\times$2), *trn*V-GCA, *trn*W-CCA, and *trn*Y-GUA
Other genes	Maturase	*mat*K
	Protease	*clp*P**
	Envelope_membrane_protein	*cem*A
	Acetyl-CoA_carboxylase	*acc*D
	C-type_cytochrome_synthesis_gene	*ccs*A
Genes of unknown function	Proteins_of_unknown_function	*ycf*1, *ycf*2($\times$2), *ycf*3**, *ycf*4, *ycf*15($\times$2)

(×N) indicates the genes that have N copies. * and ** indicate genes containing one and two introns, respectively.

### Comparative plastome analyses of *Reichardia* and *Launaea*

**Figure 3:**
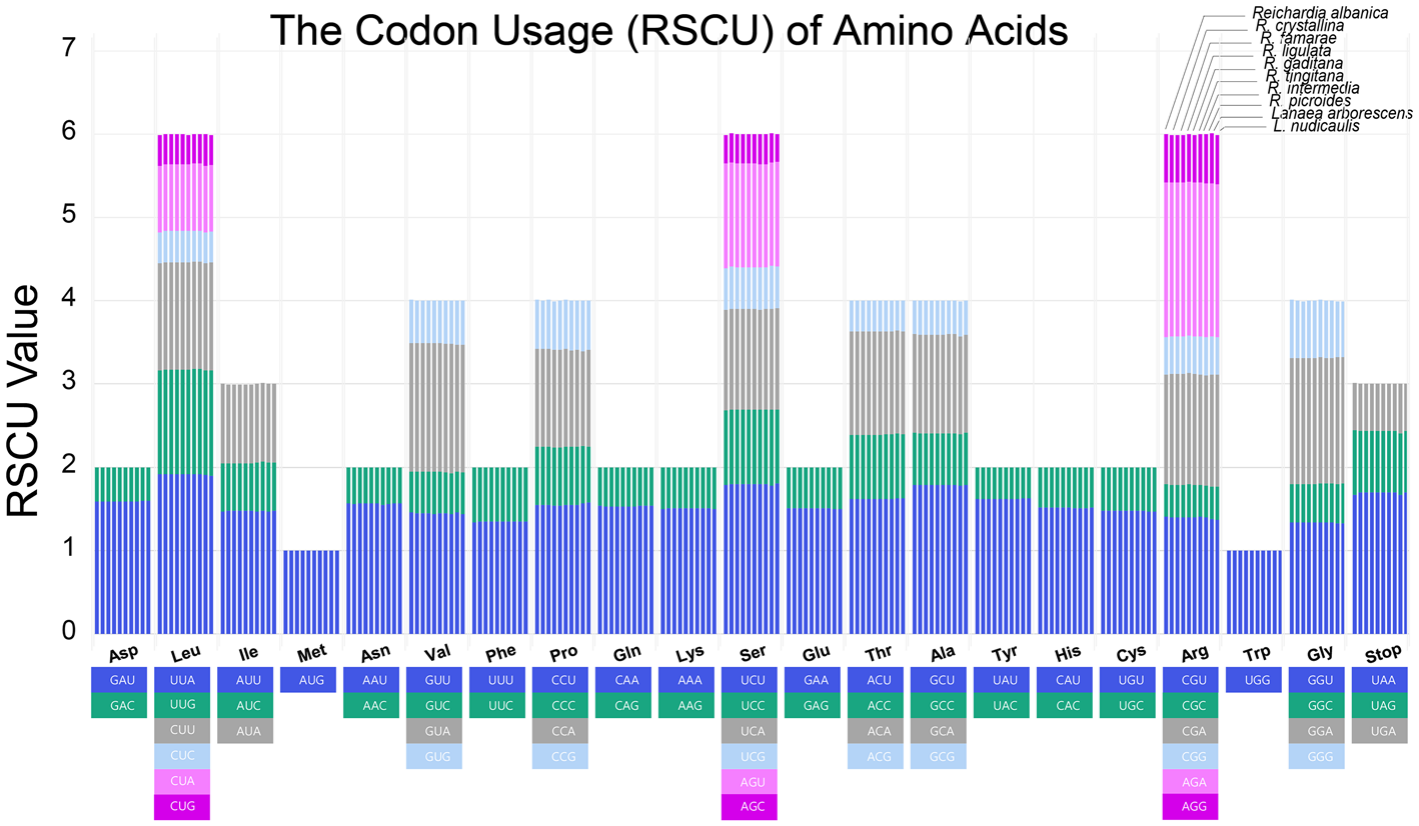
The relative synonymous codon usage (RSCU) of the protein-coding genes in chloroplast genomes of eight *Reichardia* and two *Launaea* species. The codon usages of amino acids are plotted along the x-axis, while the stacked RSCU values in each bar column are plotted along the y-axis respectively. Each amino acid contains ten clustered bar columns representing ten species; 1st column through 10th column for *R. albanica, R. crystallina, R. famarae, R. ligulata, R. gaditana, R. tingitana, R. intermedia, R. picroides, L. arborescens*, and *L. nudicaulis*.

The frequency of codon usage in the eight *Reichardia* and two *Launaea* plastomes was calculated based on protein coding gene sequences (Fig. 3 and Supplementary Table S1). The codon usage numbers in the ten *Reichardia* and *Launaea* plastomes ranged from 22,765 to 22,791, and the patterns of frequently used codons were also consistent among them except for slight variations in codon usage numbers. Within *Reichardia*, species with chromosome numbers of n = 8, except *R. intermedia*, showed lower codon usage (22,765) than species with chromosome numbers of n = 9 (*R. albanica*: 22,781) or n = 7 (*R. picroides*: 22,790), and *R. intermedia* (22,791). Codon usage values are described by relative synonymous codon usage (RSCU), which reflects how often a particular codon is used relative to the expected number of times that codon would be used in the absence of codon usage bias. All the RSCU values for each amino acid considered in this study were similar among the ten plastomes. The highest RSCU value was indicated in the usage of the UUA codon for leucine (1.9–1.92), followed by AGA for arginine (1.84–1.86), while the lowest were indicated in the usages of the AGC codon for serine (0.33–0.36) and CUG for leucine (0.35–0.38).

**Figure 4:**
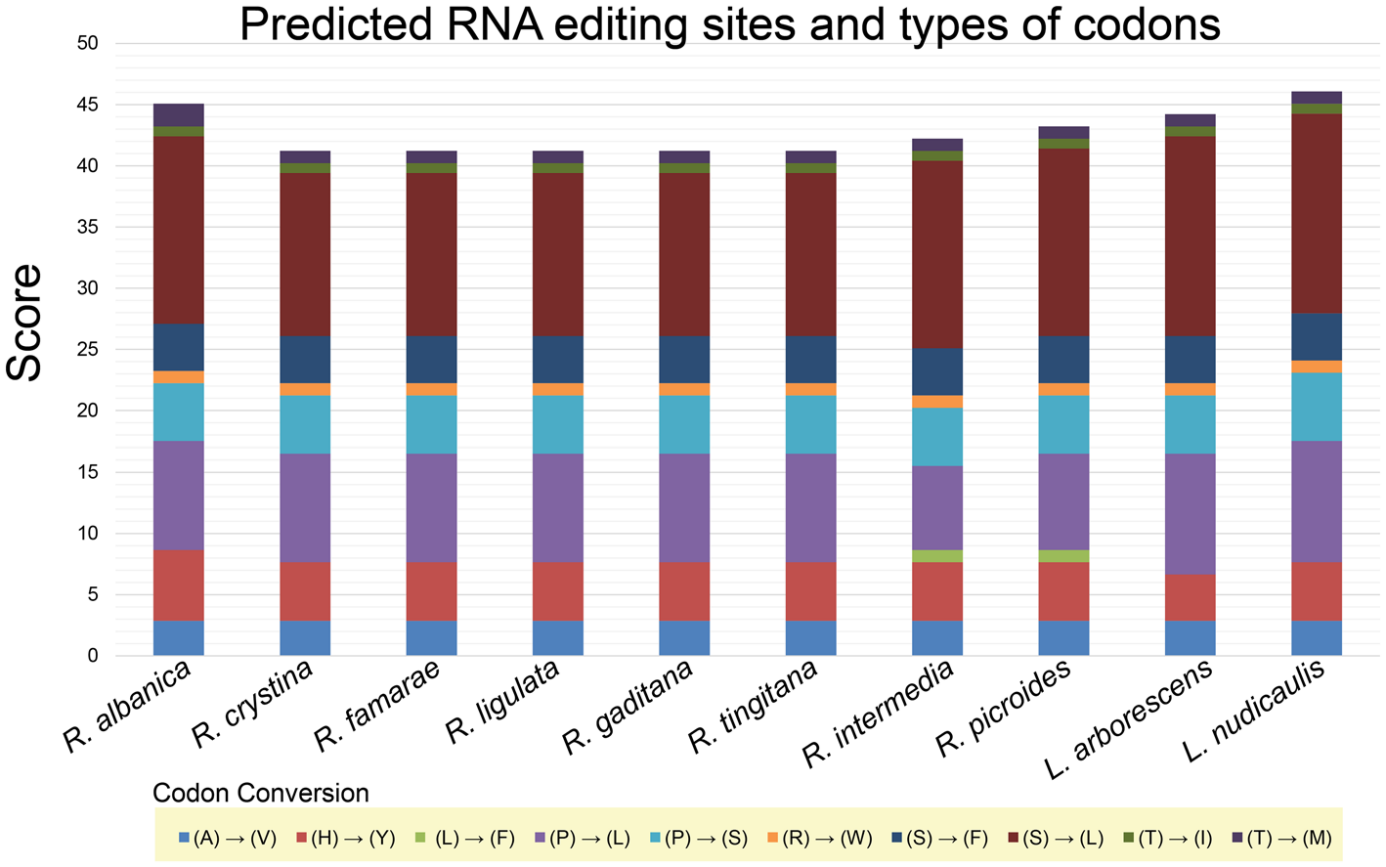
Amino acid changes in predicted RNA editing sites in ten chloroplast genomes of eight *Reichardia* and two *Launaea* species. The scores (the proportion of sites that have the same amino acid at that position) of each edit site are stacked in each bar column. Color bricks indicate RNA editing effect: Alanine to Valine, A → V; Histidine to Tyrosine, H → Y; Leucine to Phenylalanine, L → F; Proline to Phenylalanine, P → F; Proline to Leucine, P → L; Proline to Serine, P → S; Arginine to Tryptophan, R → W; Serine to Phenylalanine, S → F; Serine to Leucine, S → L; Threonine to Iso-leucine, T → I; and Threonine to Methionine, T → M.

The possible RNA editing sites predicted among the *Reichardia* and *Launaea* plastomes ranged from 43 to 48 in the 19 genes of the 35 protein-coding genes (Fig. 4 and Supplementary Table S2). We found that the RNA editing patterns across the ten *Reichardia* and *Launaea* plastomes were similar in gene location and codon conversion type of the predicted RNA editing sites, and only slight changes were observed in the number of editing sites for several codon conversions. Within *Reichardia*, all species with chromosome numbers of n = 8 showed lower numbers of predicted editing sites (43 for all species, with the exception of 44 for *R. intermedia*) than species with chromosome numbers of n = 7 (45 for *R. picroides*) and n = 9 (*R. albanica*), with 47 sites showing the highest number in the genus. Genes with predicted sites included photosynthesis-related genes (*atpA, ndhA, ndhB, ndhD, ndhF, ndhG, petB, psbF*, and *psbL*), self-replication genes (*rpl20, rpoA, rpoB, rpoC1, rpoC2, rps2*, and *rps14*), and others (*accD, ccsA*, and *matK*). We detected no RNA editing sites in *atpB, atpF, atpI, clpP, petD, petG, petL, psaB, psaI, psbB, psbE, rpl2, rpl23, rps8, rps16*, and *ycf3*. The highest numbers of potential editing sites were found in the NADH dehydrogenase genes, which was consistent with previous findings in tobacco, maize, rice, and other plants^34–37^; the *ndhB* gene was the highest at 9–10 sites, followed by the *ndhD* gene at 4–5 sites. The highest conversions in the editing frequencies of codons associated with the corresponding amino acid changes were represented by the changes from serine (S) to leucine (L) (average confidence score of 14.52), followed by proline (P) to leucine (L) (average confidence score of 8.76).

**Figure 5:**
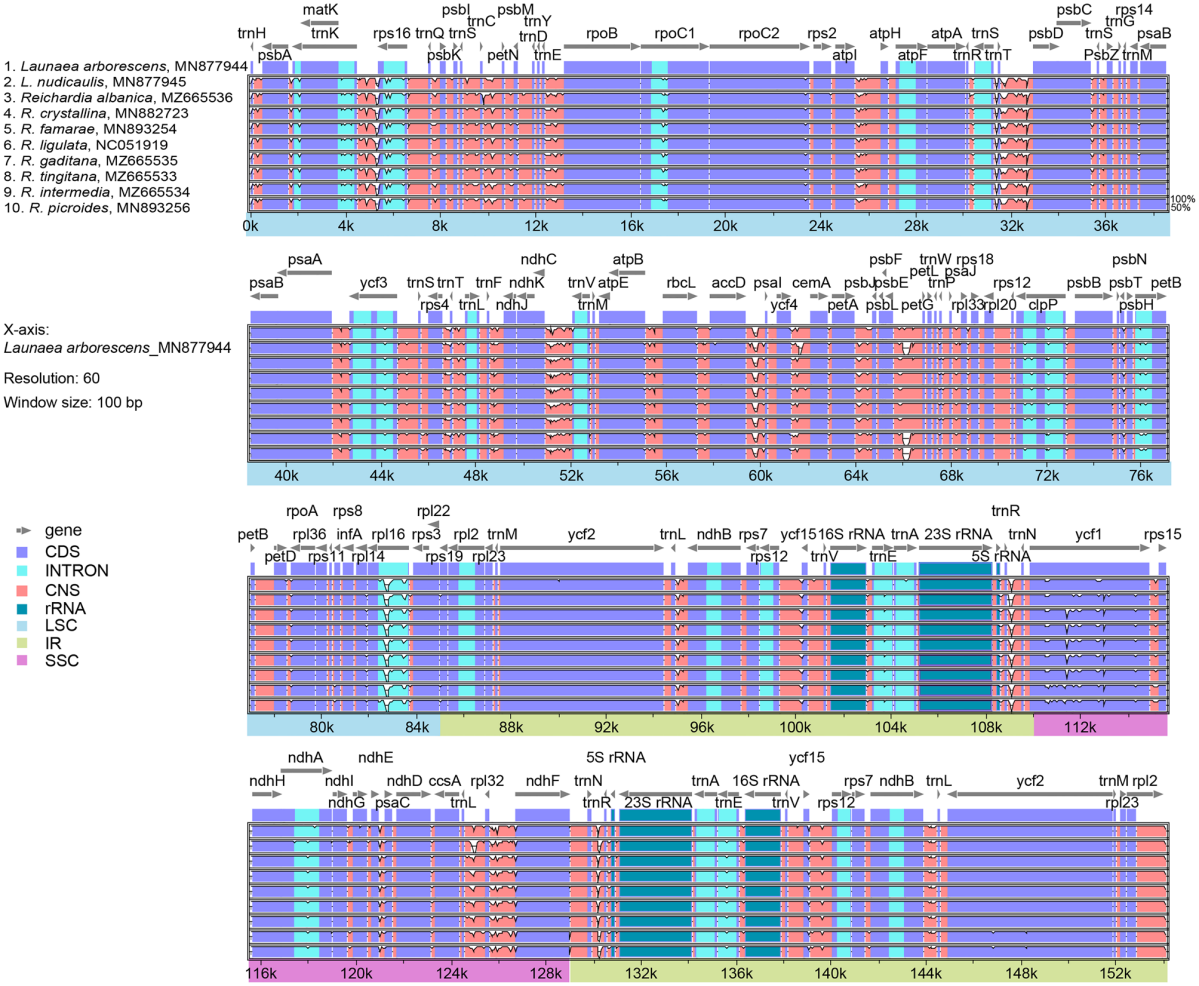
Comparison of the chloroplast genomes of eight *Reichardia* and two *Launaea* species against *L. arborescens* using mVISTA. Grey arrows indicate genes with their orientation and position. Genome regions are color-coded, as indicated.

**Figure 6:**
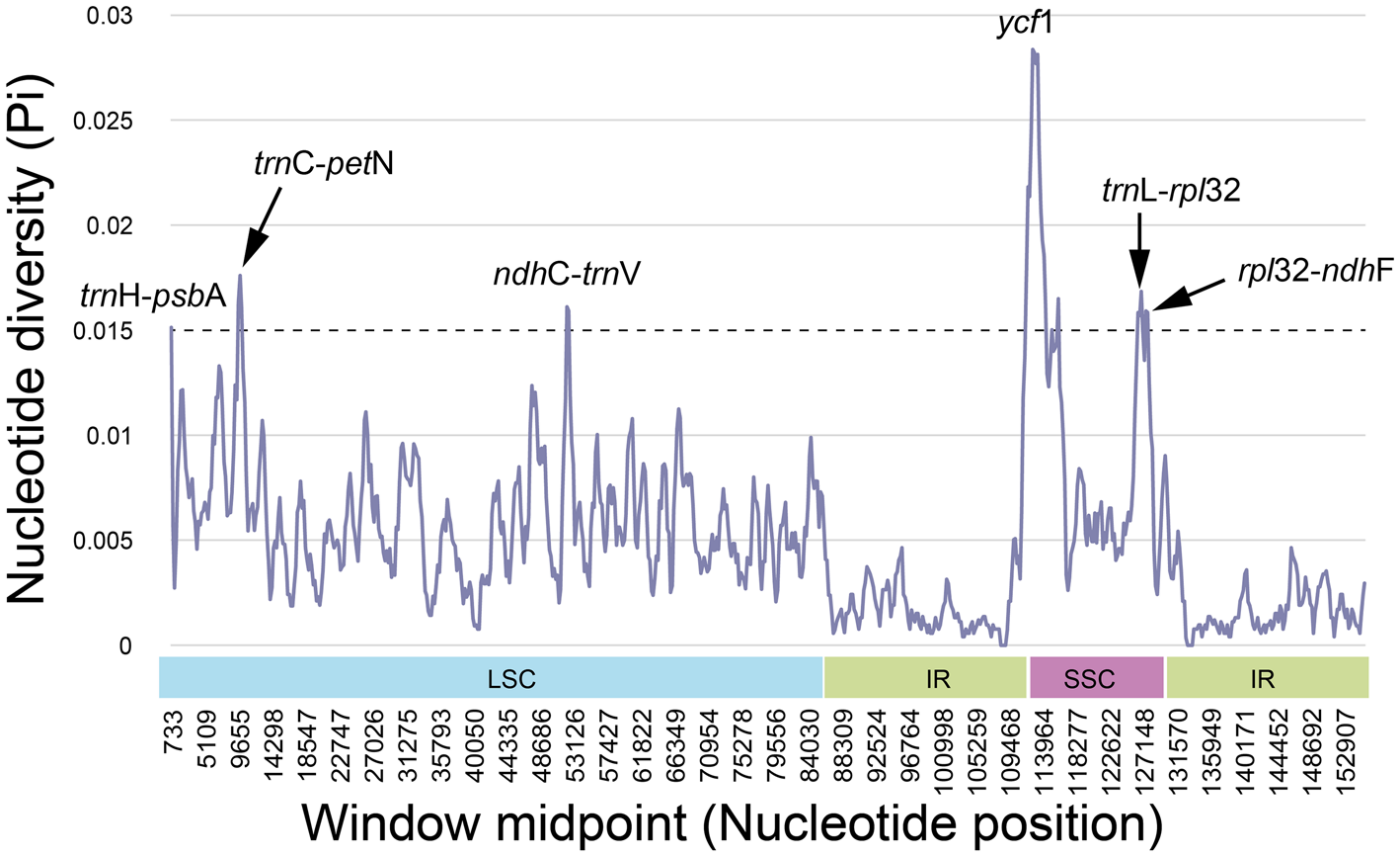
Six mutation hotspot regions were observed in eight *Reichardia* plastomes and two *Launaea* plastomes.

The divergence level of nucleotide diversity between the *Reichardia* and *Launaea* plastomes was compared using DnaSP^38^ and visualized by plotting with mVISTA^39^. The results showed a high degree of synteny and gene order conservation in the mVISTA graph (Fig. 5). The overall nucleotide diversity (Pi) of the ten plastomes was 0.00520, with 2586 polymorphic sites, ranging from 0 to 0.02836, which was higher than that of *Sonchus* species (average Pi value of 0.00283, ranging from 0 to 0.01593) belonging to the same subtribe Hyoseridinae^33^. Genetic polymorphisms in different regions of the chloroplast genome varied substantially. The SSC region, where the most variable gene, *ycf1*, was located, was the most divergent (average Pi, 0.0102), whereas the two IR regions were highly conserved (average Pi, 0.00173). Six divergence hotspots among the *Reichardia* and *Launaea* plastomes are suggested as potential chloroplast markers: five intergenic regions (*trnH-psbA, trnC-petN, ndhC-trnV, trnL-rpl32*, and *rpl32-ndhF*) and one protein-coding gene (*ycf1*) (Fig. 6).

Selective pressure in genes or genomic regions is inferred by the proportion of amino acid substitutions, and the ratio (ω = dN/dS) of nonsynonymous substitution (dN) and synonymous substitution rates (dS) has been widely used as a genomic signature of selective pressure acting on a protein-coding gene, that is, ω = 1 indicates neutral mutations, ω < 1 indicates purifying selection, and ω > 1 indicates diversifying positive selection^40^. We identified that seven genes potentially evolved under positive selection in ten *Reichardia* and *Launaea* plastomes by calculating the dN/dS ratio using various site-specific substitution models implemented in EasyCodeML (Table 3 and Supplementary Table S3)^41,42^. These genes included NADH-dehydrogenase subunit genes (*ndhB* and *ndhG*), subunit genes of photosystem I (*psaI*) and II (*psbH*), and large subunit genes of ribosomal protein (*rpl20*), *ycf2*, and *ycf15*. Positively selected sites were suggested based on the posterior probability calculated using the Bayes empirical Bayes (BEB) method^43^ with a cutoff > 0.95 and > 0.99 indicated with asterisks (* and **, respectively) in Table 3. Despite the critical importance of the genes that a plastome carries and its high conservativeness, the latest empirical evidence revealed that the variable genes potentially evolve under positive selection in the plastomes of a few other plant groups; three genes (*rps2*, *rbcL*, and *ndhG*) have been identified in *Paulownia*^44^, five (*rbcL, clpP, atpF, ycf1*, and *ycf2*) in *Panax*^45^, three (*clpP, ycf1*, and *ycf2*) in the tribe Sileneae^46^, and six (*accD, rbcL, rps3, ndhB, ndhD*, and *ndhF*) in Rosaceae^37^.

**Table 3:** Positively selected sites with dN/dS values > 1 detected in two *Launaea* and eight *Reichardia* chloroplast genomes.

Gene name	Site models	np	ln L	Model compared	LRT *p*-value	Positively selected sites
*ndhB*	M8	23	− 2072.828684	M7 vs. M8	0.250784820	87 F 0.990*, 205 S 0.990*, 210 E 0.990*, 424 G 0.990*, 428 G 0.990*, and 431 F 0.990*
	M7	21	− 2074.211844			
*ndhG*	M8	23	− 748.902533	M7 vs. M8	0.000639245	18 G 0.952* and 90 F 0.991**
	M7	21	− 756.257756			
*psaI*	M8	23	− 142.875382	M7 vs. M8	0.521237233	4 L 0.962* and 23 M 0.962*
	M7	21	− 143.526932			
*psbH*	M8	23	− 298.305462	M7 vs. M8	0.057472483	11 R 0.960* and 21 D 0.989*
	M7	21	− 301.161911			
*rpl20*	M8	23	− 523.911808	M7 vs. M8	0.384904126	25 R 0.984*, 85 L 0.984*, 110 M 0.984*, and 122 K 0.984*
	M7	21	− 524.866569			
*ycf2*	M8	23	− 9554.618258	M7 vs. M8	0.005774440	1778 I 0.984*
	M7	21	− 9559.772572			
*ycf15*	M8	23	− 264.859366	M7 vs. M8	0.408814407	23 A 0.962* and 33 R 0.962*
	M7	21	− 265.753860			

np: number of parameters in the ω distribution; ln L: log-likelihood values; LRT p-value: likelihood ratio test p-value; positive selection sites are inferred by significant posterior probability value with * > 0.95 or ** > 0.99.

### Phylogenomic analyses and ancestral state reconstructions

**Figure 7:**
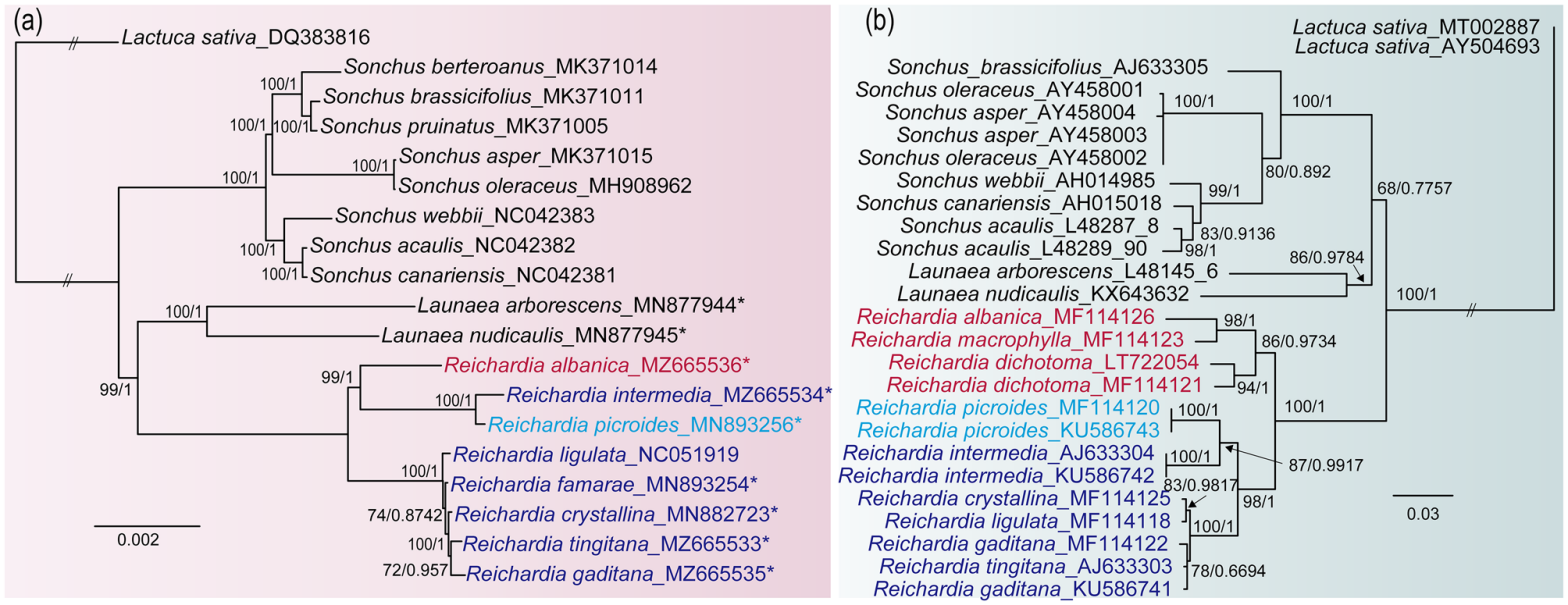
Maximum likelihood (ML) tree of *Reichardia* and closely related *Launaea* and *Sonchus* species based on (**a**) the complete plastome and (**b**) nrDNA ITS sequences. Support values are provided above and below branches (ML bootstrap value with 1000 replicates on the left and Bayesian Inference posterior probabilities on the right). “//” indicates that the branch lengths between outgroup and ingroup taxa are shortened to improve readability of trees. Newly sequenced nine chloroplast plastid genomes in this study are marked with an asterisk (*). Within *Reichardia*, the species with same chromosome numbers are colored in red for n = 9, navy blue for n = 8, and aqua blue for n = 7 chromosomes.

Phylogenetic trees were constructed using maximum likelihood (ML) and Bayesian inference (BI) methods based on the plastome and nrDNA ITS sequences to infer the phylogenetic relationships among *Reichardia* and the closely related species in the subtribe Hyoseridinae, with *Lactuca sativa* as an outgroup. The topological structures of ML phylogenetic trees constructed by IQ-TREE^47^ and BI trees by MrBayes 3.2.7a^48^ were consistent, and BI trees showed higher support values than ML trees (Fig. 7 and Supplementary Fig. S1). The best-fit evolutionary models in ML analyses were selected as “TVM + F + I” for plastome trees and “TIM3e + G4” for the nrDNA ITS tree using ModelFinder^49^ implemented in IQ-TREE. The plastome phylogenies were reconstructed using both whole plastome sequences (Fig. 7a) and 80 chloroplast protein-coding genes (Supplementary Fig. S1) of 18 representative Hyoseridinae species. The phylogenies based on plastome datasets shared the same topology and provided much greater resolution with strong bootstrap (BS) values for inter- and intra-generic relationships compared to previous studies^27,28^. The genus *Reichardia* was resolved as monophyletic and shared the most recent common ancestor with the genus *Launaea*, placing the clade of *Reichardia* and *Launaea* sister to the genus *Sonchus* s.l. Within *Reichardia*, *R. albanica* (n = 9) shared the most recent common ancestor with *R. picroides* and *R. intermedia* with chromosomal numbers of n = 7 and 8 robustly (99% or 97% BS support value in ML trees and posterior probability, PP, 1 in BI trees). The remaining five species (*R. ligulata, R. famarae, R. crystallina, R. tingitana*, and *R. gaditana*) with chromosome numbers of n = 8 formed a clade with full support (100% BS for ML and PP 1 for BI phylogenies), which was sister to the clade of n = 7, 8, and 9. On the other hand, the nrDNA ITS phylogeny showed incongruence in inter- and intra-generic relationships compared with the plastome phylogeny (Fig. 7b). Unlike plastome phylogenies, *Launaea* shared the most recent common ancestor with *Sonchus* s.l., instead of *Reichardia*; this relationship, however, was weakly supported (68% BS in ML and 0.7867 PP in BI trees). *Reichardia* was monophyletic in both nrDNA ITS ML and BI phylogenies (100% BS and 1 PP, respectively), but differed in intra-generic relationships from the plastome trees; *R. intermedia* (n = 8), and *R. picroides* (n = 7) displayed a closer affinity with the other species with a chromosome number of n = 8 (*R. ligulata, R. famarae, R. crystallina, R. tingitana*, and *R. gaditana*) than species with a chromosome number of n = 9 (*R. albanica, R. macrophylla*, and *R. dichotoma*) (98% BS in ML and 1 PP in BI trees).

**Figure 8:**
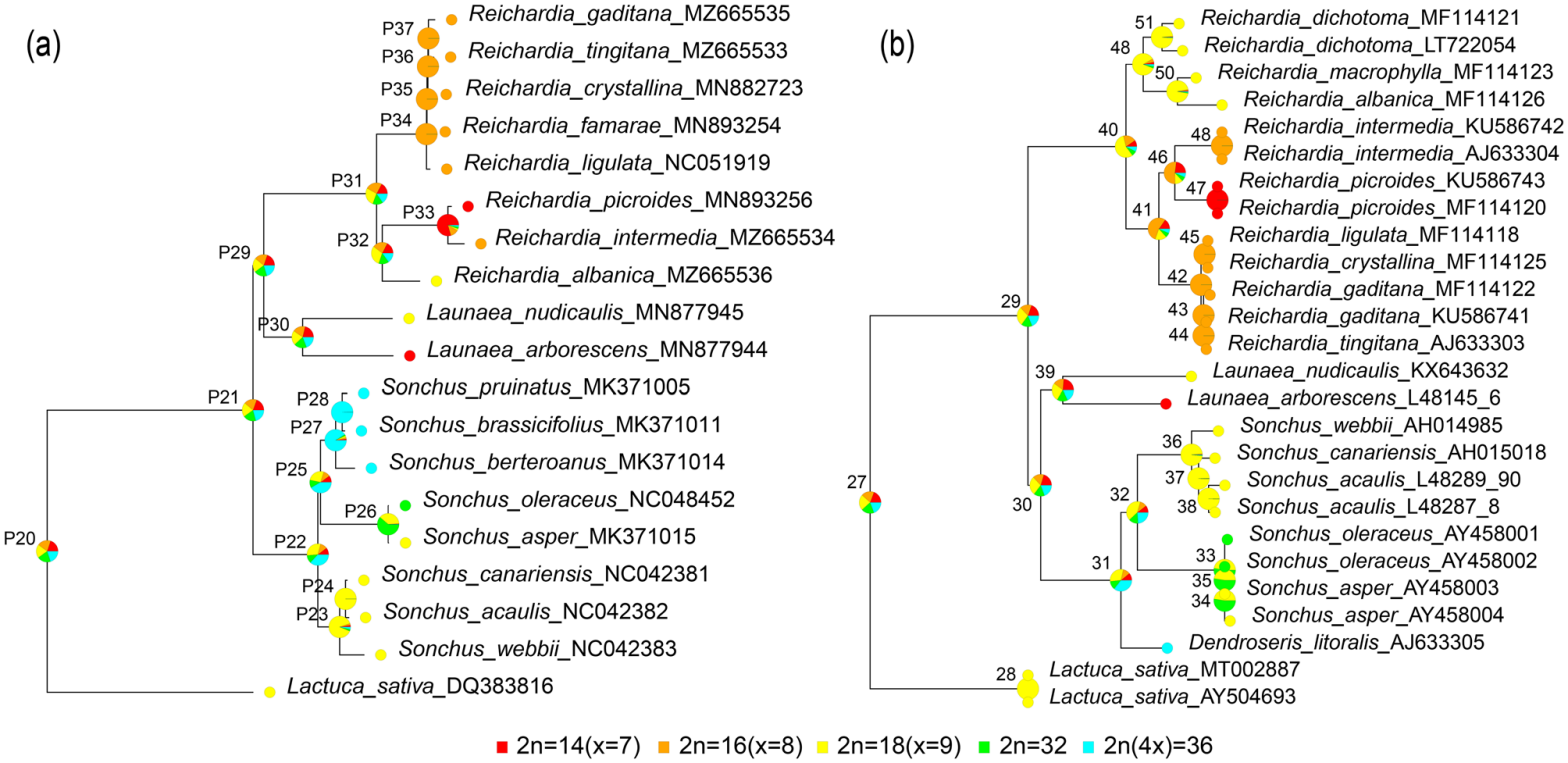
The ancestral states and shared-derived karyotypes reconstructed using MrBayes Ancestral States with R (MBASR) mapped on the ML trees based on (**a**) plastome and (**b**) nrDNA ITS sequences. The tree plots overlay state marginal likelihood pie charts at each lineage split for the probabilities of each basic chromosome numbers.

The ancestral chromosome numbers optimized on the plastome and nrDNA ITS ML phylogenies also reflected the differences between both phylogenies. The ancestral chromosome number of the most recent common ancestor (MRCA) shared by three genera, *Reichardia*, *Launaea*, and *Sonchus*, was inferred to be equivocal with 2n = 14, 16, 18, and 32, and 2n = 36 for both plastome (node P21) and nrDNA ITS (node 29) datasets (Fig. 8). Within *Reichardia*, ancestral state reconstruction using the nrDNA ITS tree suggested that the MRCA of *Reichardia* most likely had 2n = 18 (56.5% probability at node 40), suggesting a decrease in dysploidy (Fig. 8b and Supplementary Table S4). Unlike the nrDNA ITS ancestral state reconstruction, the plastome reconstruction proposed that the MRCA (node P31) with 2n = 18 (27.4%, the highest probability) evolved into two lineages: one with 2n = 16 species exclusively (*R. tingitana, R. gaditana*, and species endemic to the Canary Islands), and the other with 2n = 14 (*R. picroides*), 16 (*R. intermedia*), and 18 (*R. albanica*) species (Fig. 8a and Supplementary Table S4).

## Discussion

This study provides, for the first time, greater resolution with high support for the plastome-based inter- and intra-generic relationships among *Reichardia* and closely related genera. All three genera, *Reichardia*, *Launaea*, and *Sonchus*, were confirmed as monophyletic with strong support values (BS 100% each) in both phylogenies of full plastome sequences and concatenated sequences of plastid protein-coding genes (Fig. 7 and Supplementary Fig. S1). However, with regard to inter-generic relationships, some topological incongruences were found between cp genomes and nrDNA ITS sequences, which were in agreement with the earlier results on nrDNA ITS^25,26,28^ and cpDNA marker^27,28^ phylogenies. In both plastome based phylogenies, *Reichardia* was sister to *Launaea*, and the clade of *Reichardia* and *Launaea* was sister to *Sonchus* (Fig. 7a), while *Reichardia* was sister to the clade containing *Launaea* and *Sonchus* in nrDNA ITS phylogeny (Fig. 7b). It is likely difficult to decisively determine the inter-generic relationships in this study based on the current sampling, which included only two *Launaea* species. Given its species diversity (approximately 54 species and 10 subspecies), life form variability (perennial and annual herbs, subshrubs, cushion-forming rosette shrubs, and spinescent shrubs), and chromosome variation (n = 9, 8, 7, 6, and 5)^50^, the phylogenetic position of the genus *Launaea* remains to be clarified in future studies.

Species relationships within *Reichardia* also demonstrated incongruences between the cp genome and nrDNA ITS phylogenies. One novel finding of the current plastid phylogenomic study was that species with different chromosome numbers of n = 7 (*R. picroides*), n = 8 (*R. intermedia*), and n = 9 (*R. albanica*) were resolved in the same clade. This clade was sister to another clade with n = 8 species exclusively. In the nrDNA ITS phylogeny reconstructed previously^19^ and in this study (Fig. 7b), a clade composed of species with n = 9 chromosomes (*R. albanica, R. macrophylla*, and *R. dichotoma*) was resolved as sister to a clade of the species with chromosome numbers of n = 8 and n = 7, clearly supporting the descending dysploidy process for chromosomal evolution in *Reichardia*. One of the derived groups included two species with n = 7 and 8 (*R. picroides* and *R. intermedia*, respectively), whereas the other was exclusively comprised of five n = 8 species (*R. ligulata, R. famarae, R. crystallina, R. tingitana*, and *R. gaditana*). However, the current study strongly suggested a close relationship among n = 7, n = 9, and a representative with n = 8 (*R. intermedia*) species on both plastome-based phylogenies (Fig. 7a and Supplementary Fig. S1). According to our results, two major lineages may have diverged early from the common ancestor of the *Reichardia* species, that is, one lineage with n = 8 exclusively and the other lineage containing species with n = 9, 8, and 7. These relationships were not in agreement with a simple sequential descending dysploidy pattern from n = 9 to n = 7, as suggested by the nrDNA ITS phylogeny. The ancestral *Reichardia* karyotype was suggested to be equivocal, but n = 9 was most likely (56.5% probability at node 40; Fig. 8b and Supplementary Table S4) from the reconstruction mapped onto the nrDNA ITS ML tree. Even from the plastome tree, it was considered equivocal, but with the highest probability for n = 9 (27.4% at node P31), followed by n = 8 (24.6%), and n = 7 (17.3%). Based on these results, we can hypothesize that the common ancestor of *Reichardia* (with the most likely chromosome number of n = 9) initially diverged into two lineages: one with n = 8 species and the other including n = 9, n = 8, and n = 7 species. The hypothesis that n = 9 is the ancestral chromosome number in *Reichardia* coincides with the closely related genera *Launaea* and *Sonchus*, where this number is the most common and widely distributed^13,50,51^. Furthermore, it has been assumed as the ancestral basic chromosome number of the tribe Cichorieae^52–54^.

Given their current geographical distribution ranges, the affinity of n = 9 species with *R. intermedia* (n = 8) and *R. picroides* (n = 7) based on plastome sequences is an unexpected finding. The last two species show a wide distribution in large circum-Mediterranean regions in Africa, Asia, and Europe which hardly overlapped with those of n = 9 species and only partly with *R. dichotoma* in certain regions of West Asia (Turkey and Lebanon-Syria)^3–5^. In contrast to these two species, n = 9 species are currently found in very limited and isolated areas with a disjunctive geographical distribution pattern; Albanian endemic *R. albanica* is found only in Mount Çika in southern Albania at altitudes between 1000 and 1700 m above sea level (a.s.l.), Balkan endemic *R. macrophylla* in the central part of the Balkan Peninsula (East Dinarides) at altitudes between 500 and 1750 m a.s.l., and Western Asiatic *R. dichotoma* in Turkey, Lebanon-Syria, Iran, and North and Trans-Caucasus^7^. Considering the putative ancestral *Reichardia* karyotype (n = 9) and relic species restricted to the eastern Mediterranean^17^, it is highly plausible that *Reichardia* originated in the eastern Mediterranean. The mean divergence time based on the nrDNA ITS sequences for the two closely related genera *Launaea* and *Sonchus* was estimated to be 6.4 million years (myr)^55^. The estimated divergence time of *Reichardia* from its close relatives could be earlier than 6.4 myr based on the ITS tree topology (i.e., *Reichardia* is sister to the clade of *Launaea* and *Sonchus*) or younger than 6.4 myr on the plastome tree (i.e., *Sonchus* is sister to the clade of *Launaea* and *Reichardia*). Thus, according to the proposed divergence estimation, we can hypothesize that *Reichardia* originated around the Messinian salinity crisis in the Mediterranean basin^56^. It is plausible that after its origin in the eastern region, *Reichardia* rapidly expanded its range and diversified during dramatic climatic changes in the Mediterranean, forming n = 8 (*R. intermedia*) and n = 7 (*R. picroides*) lineages. In addition, the rapid speciation of n = 8 species in the western Mediterranean region, including species endemic to Macaronesian Islands (*R. famarae*, *R. crystallina*, and *R. ligulata*), most likely occurred after initial splitting from the n = 9 lineages. The Macaronesian Islands are well known for adaptive radiation and the diversification of explosive insular species of numerous plant lineages, including *Aeonium*^57^, *Argyranthemum*^58,59^, *Echium*^60,61^, *Tolpis*^62^ and woody *Sonchus* alliance^25–28^. The uncertain phylogenetic position of *Reichardia* relative to *Launaea* and *Sonchus* and n = 9 likely ancestral may also suggest that the common ancestor of *Reichardia* existed widely in the circum-Mediterranean region in the late Miocene and subsequently diversified during climatic turmoil, forming a relictual n = 9 lineage in the eastern Mediterranean and speciating in the western part of the Mediterranean. Furthermore, we cannot ignore the role of Quaternary climatic oscillations in shaping distribution areas, particularly in current relicts. The wide distribution range of *R. tingitana*, which roughly coincides with the paleo-geographical limits of the Mediterranean, seems to indicate, at least partially, that the expansion and diversification of this lineage may not have been recent and must have taken place prior to the present geologic configuration of the Mediterranean region. Given the phylogenetic position of *R. tingitana* (i.e., positioned in a recently derived clade), we cannot completely rule out the possibility of its recent rapid range expansion during climatic oscillation in the region.

The phylogenetic relationships among *Reichardia* species based on plastome sequences were also supported by morphological and chemotaxonomic data. Achene heteromorphy was less marked in the n = 9 species than in the other groups. *R. intermedia* and *R. picroides* showed plainly smooth inner achenes with differentiated shapes (Fig. 1c). In addition, species with purple base ligules were exclusively present in *R. tingitana*, *R. gaditana*, and the Canary Islands endemic clade. Recio et al.^24^ divided *Reichardia* species into two groups by scanning electron microscopy, in support of the findings of the present study. In group I, which contained *R. intermedia* and *R. picroides*, the outer achene surface was made up of isodiametric papillated cells, while the cylindrical/conic-truncated inner cells showed long and more or less rectangular cells without papillae. They also included *R. macrophylla* in group I, although the inner achenes did not show the same level of distinctiveness. Contrarily, group II contained n = 8 species; *R. ligulata, R. famarae, R. crystallina, R. tingitana*, and *R. gaditana* showed similar microscopic surface traits on outer and inner achenes, that is, more or less isodiametric cells, which were clearly distinguishable in shape, surface, and color (Fig. 1c; fruits of *R. tingitana*). The separation of *Reichardia* species into two major groups seems to be corroborated further by differences in chemical compositions between *R. picroides* (group I; n = 7, 8, and 9 species) and *R. tingitana* (group II; n = 8 species)^24^.

Lastly, the phylogenetic incongruence between the plastome and nrDNA ITS phylogenies could be the result of past gene flow occurring in *Reichardia* species. Hybridization and introgression often appear to be responsible for the phylogenetic incongruence between organellar and nuclear markers, as observed in many groups of plants; however, other factors, such as phylogenetic sorting or the retention of ancestral polymorphisms, could also play a role in generating incongruences^63,64^. The *R. intermedia* and *R. picroides* group shared the most recent common ancestor with n = 9 species in plastome-based phylogenies with strong support values (BS 99% or 97% in ML and PP 1 in BI trees) (Fig. 7a and Supplementary Fig. S1), whereas they were more closely related to the n = 8 group of species in the nrDNA ITS phylogeny (BS 98% in ML and PP 1 in BI trees) (Fig. 7b). Considering the wide distribution range of both groups in large circum-Mediterranean regions in Africa, Asia, and Europe, they may easily overlap in terms of distribution. Therefore, it is plausible that *R. intermedia* and *R. picroides* captured the chloroplast genome from the ancient n = 9 group with a much broader distribution, resulting in a phylogenetic incongruence between the cpDNA and ITS phylogeny. Several cases of hybridization among *Reichardia* species have been documented, further supporting the likelihood of this hypothesis, including *R*. × *baetica* Gallego & Talavera (*R. tingitana* × *R. intermedia*), *R*. x *canariensis* Gallego & Talavera (*R. tingitana* × *R. ligulata*), and *R*. × *sventenia* Gallego & Talavera (*R. tingitana* × *R. famarae*)^6^. While the *R*. × *sventenia* and *R.* × *canariensis* cases are examples of hybridization between widely distributed *R. tingitana* and the locally restricted species endemic to the Canary Islands, the hybrid origin of *R*. × *baetica* occurred between two divergent n = 8 lineages with a much wider geographical distribution.

In conclusion, we found highly conserved plastomes, including gene order and content, in *Reichardia* and *Launaea*. We achieved the first full resolution regarding inter and intra-generic phylogenetic relationships based on the complete plastome sequences of most *Reichardia* species with variable chromosome numbers (n = 7, 8, and 9). The plastid phylogenomics strongly suggested the early divergence of two major lineages of *Reichardia*, one with a group of species with exclusively n = 8 chromosome numbers and the other with n = 9, 8, and 7 species together. Although the plastome-based relationships were not in full agreement with the stepwise descending dysploid hypothesis in *Reichardia*, the ancestral *Reichardia* karyotype was highly likely to be n = 9 species. It is necessary to utilize whole nuclear genome data and a thorough synteny analysis to elucidate convincingly the species relationships and chromosomal evolution within the genus *Reichardia*. Based on thorough characterization and comparative analyses of the plastomes, we discovered several informative mutation hotspots, which will increase the efficiency and feasibility of phylogenetic reconstruction among *Reichardia* and closely related genera.

## Methods

### Plant sampling, DNA isolation, and plastome sequencing/annotation

Plant materials of *Reichardia* and *Launaea* species were collected in the field (see plant sources in^25,26,28^), except for *R. albanica*, which was obtained from herbarium specimens (voucher number 56321, APP). Total genomic DNA was isolated using CTAB^65^ and a DNeasy Plant Mini Kit (Qiagen, Carlsbad, CA, USA) following the manufacturer’s protocol. An Illumina paired-end (PE) genomic library was constructed and sequenced using the Illumina HiSeq platform (Illumina, Inc., San Diego, CA, USA) at Macrogen Corporation (Seoul, Korea). Sequence reads of the chloroplast genomes were assembled using the de novo genomic assembler Velvet 1.2.10^66^. Annotation was performed using Geneious R10 (Biomatters, Auckland, New Zealand)^67^ and ARAGORN v1.2.36^68^. The annotated plastome sequences were deposited in GenBank under the accession numbers listed in Table 1: MZ665536 for *R. albanica*, MN882723 for *R. crystallina*, MN893254 for *R. famarae*, MZ665535 for *R. gaditana*, MZ665534 for *R. intermedia*, MN893256 for *R. picroides*, MZ665533 for *R. tingitana*, MN877944 for *L. arborescens*, and MN877945 for *L. nudicaulis*. *R. ligulata* (GenBank accession number NC051919) was obtained from our previous study^33^. The annotated GenBank (NCBI, Bethesda, MD, USA) format sequence file was used to draw a circular plastid genome map (Fig. 1a) using the OGDRAW software v1.2 (CHLOROBOX)^69^.

### Comparative plastome analyses

We performed several comparative plastome analyses of the eight *Reichardia*, representing variable chromosomal numbers. The analyses also included two related *Launaea* plastomes (*L. arborescens* and *L. nudicaulis*). The codon usage frequency was calculated using MEGA7^70^ with the relative synonymous codon usage (RSCU) value, which is the relative frequency of occurrence of the synonymous codon for a specific amino acid. The online program predictive RNA editor for plants (PREP) suite^71^ was used to predict the potential RNA editing sites for annotated protein-coding genes with 35 reference genes available with known edit sites, based on a cutoff value of 0.8 (suggested as optimal for PREP-Cp). The overall sequence divergence was estimated using the Lagan alignment mode^72^ in mVISTA^39^. The nucleotide diversity (Pi) was calculated using sliding window analysis (window length = 1000 bp and step size = 200 bp, excluding sites with alignment gaps) to detect the most divergent regions (i.e., mutation hotspots) in DnaSP^38^. To evaluate the natural selection pressure in the protein-coding genes of the ten plastomes, site-specific models implemented in EasyCodeML^41^ were used in the preset running mode based on CodeML algorithms^42^. Seven codon substitution models with heterogeneous ω values across sites were investigated and compared to detect positively selected sites based on likelihood ratio tests (M0, M1a, M2a, M3, M7, M8, and M8a).

### Phylogenetic analyses and ancestral state reconstructions

The phylogenetic positions of the newly sequenced plastomes of *Reichardia* and *Launaea* assembled in this study were investigated in the context of their relationships with closely related species in the subtribe Hyoseridinae, including the outgroup species *Lactuca sativa* from the same tribe (Cichorieae). We further compared the plastome phylogeny with the nrDNA ITS phylogeny to explore phylogenetic incongruence and gain insights into chromosome evolution. To reconstruct the plastome phylogenies, we analyzed 19 plastomes of major genera of the subtribe Hyoseridinae and one outgroup based on whole plastome sequences, as well as concatenated sequences of 80 common protein-coding genes, including only nine newly sequenced species and downloaded plastomes from GenBank (Table 1 and Supplementary Table S5). The nrDNA ITS phylogeny was also reconstructed using the retrieved sequences of 26 accessions from GenBank as specified in Supplementary Table S5, which were selected similarly as the taxa in the plastome phylogeny for the species belonging to the genera *Reichardia*, *Launaea*, and *Sonchus* s.l. in the subtribe Hyoseridinae. The plastome and nrDNA ITS sequences were aligned using MAFFT v. 7^73^, and phylogenetic trees were constructed using IQ-TREE v. 1.4.2, with 1000 bootstrap replicates^47^ for ML and MrBayes v. 3.2.7a^48^ for BI. The best-fit evolutionary models in ML trees were scored according to Bayesian information criterion (BIC) scores and weights by testing 88 DNA models of ModelFinder^49^ implemented in IQ-TREE v. 1.4.2. The evolutionary model of GTR + I + Г (General Time Reversible substitution model with a proportion of invariable sites and a gamma-distributed rate across sites) was selected for Bayesian inference. The Markov chain Monte Carlo simulation (MCMC) had a length of 4,000,000 generations, sampling every 100 generations, for both plastome and nrDNA ITS phylogeny. The average standard deviation of the split frequencies reached below 0.01, which indicates that two runs reached stationarity. The first 25% of samples were discarded as burn-in, and the remaining samples were retained for the construction of a 50% majority-rule consensus tree with clade frequencies.

The reconstructed ML trees for the plastome and nrDNA ITS sequences were used as phylogenetic hypotheses to perform ancestral state reconstruction of chromosome numbers using the R toolkit^74^ and MrBayes ancestral states with R (MBASR)^75^. The coded trait scores of chromosome numbers of each taxon were mapped onto both phylogenies, and the ancestral states and shared-derived karyotypes were inferred using MrBayes 3.2.7a^48^. The parameters in the analyses were set to “character.type = unordered” and “n.samples = 500”.

## Supplementary Information

Supplementary Legends.

Supplementary Figure S1.

Supplementary Table S1.

Supplementary Table S2.

Supplementary Table S3.

Supplementary Table S4.

Supplementary Table S5.

## Data Availability

The plastome datasets sequenced and analyzed in the current study are available from GenBank under the accession numbers specified in Table 1.

## References

[1] Axelrod DI (1975). Evolution and biogeography of Madrean-Tethyan sclerophyll vegetation. Ann. Mo. Bot. Gard..

[2] Takhtajan, A. Floristic regions of the world. 522 (University California Press, 1986).

[3] Kilian, N., Hand, R. & Raab-Straube, E. von (general editors) (continuously updated). Cichorieae Systematics Portal. http://cichorieae.e-taxonomy.net/portal/, accessed (Accessed on 2 Sept 2021) (2009).

[4] POWO. Plants of the World Online. Facilitated by the Royal Botanic Gardens, Kew. http://www.plantsoftheworldonline.org/taxon/urn:lsid:ipni.org:names:10818-1 (Accessed 26 Aug 2021).

[5] GBIF. Global Biodiversity Information Facility. *Reichardia* Roth. https://www.gbif.org/es/species/2965909 (Accessed 24 Dec 2021).

[6] Gallego MJ, Talavera S, Silvestre S (1980). Revision del género *Reichardia* Roth (Compositae). Lagascalia.

[7] Conti F, Niketić M, Vukojičić S, Siljak-Yakovlev S, Barina Z, Lakušić D (2015). A new species of *Reichardia* (Asteraceae, Cichorieae) from Albania and re-evaluation of *R. macrophylla*. Phytotaxa.

[8] Bramwell D, Bramwell Z (2001). Wild flowers of the Canary Islands.

[9] Moreno, J. C. Lista Roja de la flora vascular española. Actualización con los datos de la Adenda 2010 al Atlas y Libro Rojo de la Flora Vascular Amenazada, Dirección General de Conservación de la Naturaleza y Sociedad Española de Biología de la Conservación de Plantas: Madrid, Spain, 1–46 (2011).

[10] Index to Chromosome numbers in Asteraceae. http://www.lib.kobe-u.ac.jp/infolib/meta_pub/sresult (Accessed 9 Sept 2021).

[11] Löve Á, Kjellqvist E (1974). Cytotaxonomy of Spanish plants. IV. Dicotyledons: Ceslpiniaceae-Asteraceae. Lagascalia.

[12] Van Loon JC (1974). A cytological investigation of flowering plants from the Canary Islands. Acta. Bot. Neerl..

[13] Gallego MJ (1980). Estudio cariológico de las especies españolas del género *Reichardia* Roth (Compositae). Lagascalia.

[14] Siljak-Yakovlev S (1981). Analyse comparative des caryotypes de deux especes du genre *Reichardia* Roth (*R. macrophylla* Vis. & Pancic et *R. **picroides* (L.) Roth) et leur relation taxonomique. Caryologia.

[15] Kamari G, Felber F, Garbari F (1998). Mediterranean chromosome number reports-8, Reports (936–940) by José A, Mejías. Fl. Medit..

[16] Kamari G, Blanché C, Siljak-Yakovlev S (2012). Mediterranean chromosome number reports-22. Fl. Medit..

[17] Ritter-Studnička H (1967). Reliktgesellschaften auf Dolomitböden in Bosnien und der Hercegovina. Plant Ecol..

[18] Fernandes, A. & Queirós, M. Contribution à la connaissance cytotaxonomyque des Spermatophytes du Portugal. II. Compositae. *Soc. Brot. Bol*. 5–121 (1971).

[19] Siljak-Yakovlev S, Godelle B, Zoldos V, Vallès J, Garnatje T, Hidalgo O (2017). Evolutionary implications of hetero-chromatin and rDNA in chromosome number and genome size changes during dysploidy: A case study in *Reichardia* genus. PLoS ONE.

[20] Rieseberg LH (2001). Chromosomal rearrangements and speciation. Trends Ecol. Evol..

[21] Levin DA (2002). The Role of Chromosomal Change in Plant Evolution.

[22] Schubert I (2007). Chromosome evolution. Curr. Opin. Pl. Evol..

[23] Vallès J (2013). Genome size variation and evolution in the family Asteraceae. Caryologia.

[24] Recio MC (1992). Phenolics of *Reichardia* and their taxonomic implications. Biochem. Syst. Ecol..

[25] Kim S-C, Crawford DJ, Jansen RK (1996). Phylogenetic relationships among the genera of the subtribe Sonchinae (Asteraceae): Evidence from ITS sequences. Syst. Bot..

[26] Kim S-C, Crawford DJ, Francisco-Ortega J, Santos-Guerra A (1996). A common origin for woody *Sonchus* and five related genera in the Macaronesian islands: Molecular evidence for extensive radiation. P. Natl. Acad. Sci. USA.

[27] Kim S-C, Crawford DJ, Jansen RK, Santos-Guerra A (1999). The use of a non-coding region of chloroplast DNA in phylogenetic studies of the subtribe Sonchinae (Asteraceae: Lactuceae). Plant Syst. Evol..

[28] Kim SC, Lee C, Mejías JA (2007). Phylogenetic analysis of chloroplast DNA *matK* gene and ITS of nrDNA sequences reveals polyphyly of the genus *Sonchus* and new relationships among the subtribe Sonchinae (Asteraceae: Cichorieae). Mol. Phylogenet. Evol..

[29] Whitton J, Wallace RS, Jansen RK (1995). Phylogenetic relationships and patterns of character change in the tribe Lactuceae (Asteraceae) based on chloroplast DNA restriction site variation. Can. J. Bot..

[30] Kilian N, Gemeinholzer B, Lack HW, Funk VA, Susanna A, Stuessy TF, Bayer RJ (2019). Cichorieae. Systematics, evolution, and biogeography of Compositae.

[31] Daniell H, Lin CS, Yu M, Chang WJ (2016). Chloroplast genomes: Diversity, evolution, and applications in genetic engineering. Genome Biol..

[32] Parks M, Cronn R, Liston A (2009). Increasing phylogenetic resolution at low taxonomic levels using massively parallel sequencing of chloroplast genomes. BMC Biol..

[33] Cho M-S, Kim SH, Yang J, Crawford DJ, Stuessy TF, López-Sepúlveda P, Kim S-C (2020). Plastid phylogenomics of *Dendroseris* (Cichorieae, Asteraceae): Insights into structural organization and molecular evolution of an endemic lineage from the Juan Fernández Islands. Front. Plant Sci..

[34] Corneille S, Lutz K, Maliga P (2000). Conservation of RNA editing between rice and maize plastids: Are most editing events dispensable?. Mol. Gen. Genet..

[35] Kim S-H, Yang JY, Park JS, Yamada T, Maki M, Kim S-C (2019). Comparison of whole plastome sequences between thermogenic skunk cabbage *Symplocarpus*
*renifolius* and nonthermogenic *S.*
*nipponicus* (Orontioideae, Araceae) in East Asia. Int. J. Mol. Sci..

[36] Tsudzuki T, Wakasugi T, Sugiura M (2001). Comparative analysis of RNA editing sites in higher plant chloroplasts. J. Mol. Evol..

[37] Yang JY, Kang GH, Pak JH, Kim S-C (2020). Characterization and comparison of two complete Plastomes of Rosaceae species (*Potentilla **dickinsii* var. *glabrata* and *Spiraea **insularis*) endemic to Ulleung Island, Korea. Int. J. Mol. Sci..

[38] Librado P, Rozas J (2009). DnaSP v5: A software for comprehensive analysis of DNA polymorphism data. Bioinformatics.

[39] Frazer KA, Pachter L, Poliakov A, Rubin EM, Dubchak I (2004). VISTA: Computational tools for comparative genomics. Nucleic Acids Res..

[40] Yang Z, Nielsen R, Goldman N, Pedersen AMK (2000). Codon-substitution models for heterogeneous selection pressure at amino acid sites. Genetics.

[41] Gao F, Chen C, Arab DA, Du Z, He Y, Ho SYW (2019). EasyCodeML: A visual tool for analysis of selection using CodeML. Ecol. Evolut..

[42] Yang Z (1997). PAML: A program package for phylogenetic analysis by maximum likelihood. Bioinformatics.

[43] Yang Z, Wong WS, Nielsen R (2005). Bayes empirical Bayes inference of amino acid sites under positive selection. Mol. Biol. Evol..

[44] Li P, Lou G, Cai X, Zhang B, Cheng Y, Wang H (2020). Comparison of the complete plastomes and the phylogenetic analysis of *Paulownia* species. Sci. Rep..

[45] Jiang P, Shi FX, Li MR, Liu B, Wen J, Xiao HX, Li LF (2018). Positive selection driving cytoplasmic genome evolution of the medicinally important ginseng plant genus *Panax*. Front. Plant Sci..

[46] Sloan DB, Triant DA, Forrester NJ, Bergner LM, Wu M, Taylor DR (2014). A recurring syndrome of accelerated plastid genome evolution in the angiosperm tribe *Sileneae* (Caryophyllaceae). Mol. Phylogenet. Evol..

[47] Nguyen L-T, Schmidt HA, von Haeseler A, Minh BQ (2015). IQ-TREE: A fast and effective stochastic algorithm for estimating maximum-likelihood phylogenies. Mol. Biol. Evol..

[48] Ronquist F (2012). MRBAYES 3.2: Efficient Bayesian phylogenetic inference and model selection across a large model space. Syst. Biol..

[49] Kalyaanamoorthy S, Minh BQ, Wong TK, von Haeseler A, Jermiin LS (2017). ModelFinder: Fast model selection for accurate phylogenetic estimates. Nat. Methods.

[50] Kilian N (1997). Revision of *Launaea* Cass. (Compositae, Lactuceae, Sonchinae). Englera.

[51] Mejías JA, Andrés C (2004). Karyological studies in Iberian *Sonchus* (Asteraceae: Lactuceae): *S. oleraceus*, *S. microcephalus* and *S. asper* and a general discussion. Folia Geobot..

[52] Stebbins GL, Jenkins JA, Walters MS (1953). Chromosomes and phylogeny in the Compositae, tribe Cichorieae. Univ. Calif. Publ. bot..

[53] Tomb AS, Heywood VH, Harborne JB, Turner BL (2013). Lactuceae-systematic review. The biology and chemistry of the Compositae.

[54] Tomb AS, Chambers KL, Kyhos DW, Powell AM, Raven PH (1978). Chromosome numbers in the Compositae. XIV. Lactuceae. Am. J. Bot..

[55] Tremetsberger K, Gemeinholzer B, Zetzsche H, Blackmore S, Kilian N, Talavera S (2013). Divergence time estimation in *Cichorieae* (Asteraceae) using a fossil-calibrated relaxed molecular clock. Org. Divers. Evol..

[56] Krijgsman W, Hilgen FJ, Raffi I, Sierro FJ, Wilson DS (1999). Chronology, causes and progression of the Messinian salinity crisis. Nature.

[57] Jorgensen TH, Olesen JM (2001). Adaptive radiation of island plants: Evidence from *Aeonium* (Crassulaceae) of the Canary Islands. Perspect. Plant Ecol. Evol. Syst..

[58] Francisco-Ortega J, Jansen RK, Santos-Guerra A (1996). Chloroplast DNA evidence of colonization, adaptive radiation, and hybridization in the evolution of the Macaronesian flora. Proc. Natl. Acad. Sci. USA.

[59] Imamura R, Santos-Guerra A, Kondo K (2015). A molecular phylogenetic relationship of certain species of *Argyranthemum* found in the Canary Islands of Spain on the basis of the internal transcribed spacer (ITS). Chromosome Bot..

[60] Böhle UR, Hilger HH, Martin WF (1996). Island colonization and evolution of the insular woody habit in *Echium* L. (Boraginaceae). Proc. Natl. Acad. Sci. USA.

[61] Garcia-Maroto F, Manas-Fernández A, Garrido-Cárdenas JA, López Alonso D, Guil-Guerrero JL, Guzmán B, Vargas P (2009). D6-Desaturase sequence evidence for explosive Pliocene radiations within the adaptive radiation of Macaronesian *Echium* (Boraginaceae). Mol. Phylogenet. Evol..

[62] Gruenstaeudl M, Santos-Guerra A, Jansen RK (2013). Phylogenetic analyses of *Tolpis* Adans. (Asteraceae) reveal patterns of adaptive radiation, multiple colonization and interspecific hybridization. Cladistics.

[63] Wendel JF, Doyle JJ, Soltis DE, Soltis PS, Doyle JJ (1998). Phylogenetic incongruence: window into genome history and molecular evolution. Molecular systematics of plants II DNA Sequencing.

[64] Rieseberg LH, Whitton J, Linder CR (1996). Molecular marker incongruence in plant hybrid zones and phylogenetic trees. Acta Bot. Neerl..

[65] Doyle JJ, Doyle JL (1987). A rapid DNA isolation procedure for small quantities of fresh leaf tissue. Phytochem. Bull..

[66] Zerbino DR, Birney E (2008). Velvet: Algorithms for de novo short read assembly using de Bruijn graphs. Genome Res..

[67] Kearse M (2012). Geneious Basic: An integrated and extendable desktop software platform for the organization and analysis of sequence data. Bioinformatics.

[68] Laslett D, Canback B (2004). ARAGORN, a program to detect tRNA genes and tmRNA genes in nucleotide sequences. Nucleic Acids Res..

[69] Lohse M, Drechsel O, Bock R (2009). Organellar genome DRAW (OGDRAW): A tool for the easy generation of high-quality custom graphical maps of plastid and mitochondrial genomes. Curr. Genet..

[70] Kumar S, Stecher G, Tamura K (2016). MEGA7: Molecular evolutionary genetics analysis v7.0 for bigger datasets. Mol. Biol. Evol..

[71] Mower JP (2009). The PREP suite: Predictive RNA editors for plant mitochondrial genes, chloroplast genes and user-defined alignments. Nucleic Acids Res..

[72] Brudno M, Do CB, Cooper GM, Kim MF, Davydov E, Green ED, Sidow A, Batzoglou S (2003). NISC comparative sequencing program. LAGAN and multi-LAGAN: Efficient tools for large-scale multiple alignment of genomic DNA. Genome Res..

[73] Katoh K, Standley DM (2013). MAFFT multiple sequence alignment software version 7: Improvements in performance and usability. Mol. Biol. Evol..

[74] R Development Core Team. R: A language and environment for statistical computing. http://www.R-project.org/ (Accessed 21 Oct 2021) (R Foundation for Statistical Computing, 2005).

[75] Heritage S (2021). MBASR: Workflow-simplified ancestral state reconstruction of discrete traits with MrBayes in the R environment. bioRxiv.

