# Supplementary material for: Phylogenomic insight into dysploidy, speciation, and plastome evolution of a small Mediterranean genus Reichardia (Cichorieae; Asteraceae)
Source: Sci Rep. 2022 Jun 30;12:11030. doi: 10.1038/s41598-022-15235-1 (PMC9247168; doi:10.1038/s41598-022-15235-1)
Supplement: Supplementary file 1 — Supplementary Legends. [file 41598_2022_15235_MOESM1_ESM.docx]

**Supplementary Materials**

Supplementary Figure S1. Maximum likelihood tree of *Reichardia* and closely related species from *Launaea* and *Sonchus* based on concatenated sequences of plastid protein-coding genes only. Support values are provided above and below branches (ML bootstrap value with 1000 replicates on the left and Bayesian Inference posterior probabilities on the right). Nine newly sequenced chloroplast plastid genomes are marked with an asterisk (*). Within *Reichardia*, the species with same basic chromosome numbers are colored in red for n = 9, navy blue for n = 8, and aqua blue for n = 7 chromosomes.

Supplementary Table S1. Codon-anticodon recognition pattern and codon usage for the chloroplast genomes of eight *Reichardia* (*R. albanica*, *R. crystallina*, *R. famarae*, *R. gaditana*, *R. intermedia*, *R. picroide*s, and *R. tingitana*) and two *Launaea* (*L. arborescens* and *L. nudicaulis*) species.

Supplementary Table S2. Predicted RNA editing sites in the chloroplast genomes of eight *Reichardia* and two *Launaea* species.

Supplementary Table S3. Results of EasyCodeML analyses of potentially evolving sites under positive selection in the chloroplast genomes of eight *Reichardia* and two *Launaea* species.

Supplementary Table S4. Results of MBASR (MrBayes Ancestral States with R) analyses based on plastomes and nrDNA ITS sequences for *Reichardia* and closely related species.

Supplementary Table S5. Accession numbers of plastomes and nrDNA ITS sequences downloaded from GenBank for the reconstruction of phylogenetic trees of *Reichardia* and closely related species.
