# Supplementary figures and images for: Phylogenomic insight into dysploidy, speciation, and plastome evolution of a small Mediterranean genus Reichardia (Cichorieae; Asteraceae)
Source: Sci Rep. 2022 Jun 30;12:11030. doi: 10.1038/s41598-022-15235-1 (PMC9247168; doi:10.1038/s41598-022-15235-1)

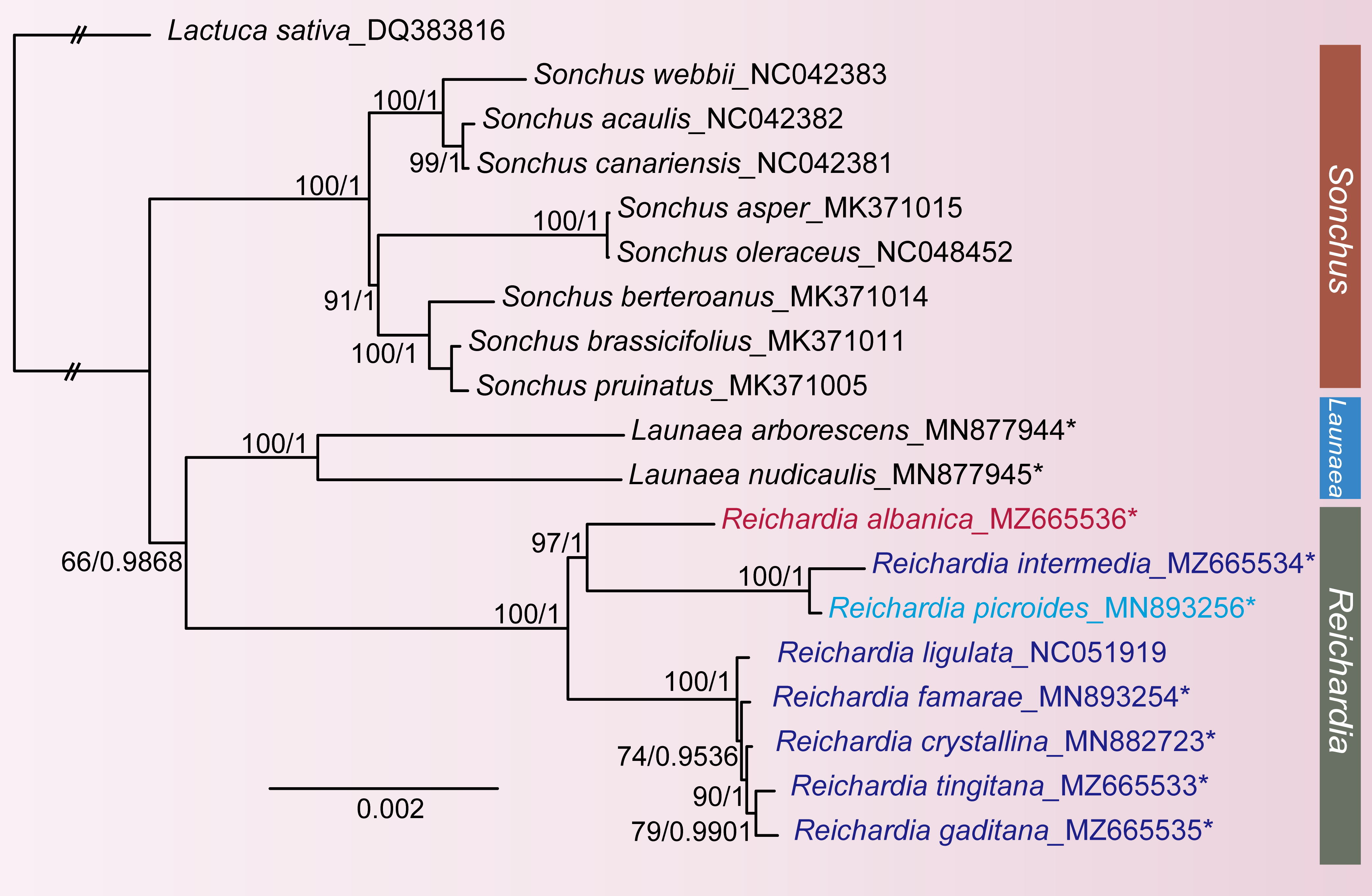

Supplement: Supplementary file 2 — Supplementary Figure S1. [file 41598_2022_15235_MOESM2_ESM.tif]
